# Undulant Fevers and Bitemporal Headaches: A Clinical Presentation of Human Brucellosis

**DOI:** 10.7759/cureus.21895

**Published:** 2022-02-04

**Authors:** Pirapon L Chaidarun, Akil H Hutchinson

**Affiliations:** 1 Internal Medicine, NewYork-Presbyterian Brooklyn Methodist Hospital, Brooklyn, USA

**Keywords:** pancytopenia, nausea, headache, fever, zoonotic, bacteremia, infectious disease, global health, brucella, brucellosis

## Abstract

Brucellosis is a common zoonotic infection endemic to certain areas of the Mediterranean, Middle East, Central America, and Sub-Saharan Africa. We present a case of brucellosis in a patient who recently traveled to Grenada and returned to the United States with a wide degree of symptoms. This case explores the etiology, clinical presentation, investigation, and treatment of brucellosis. Though a patient’s clinical presentation may be non-specific, the recognition of potential etiologies may aid in empirically treating the infection prior to laboratory confirmation.

## Introduction

Brucellosis (also coined Mediterranean fever, Malta fever, or undulant fever) is transmitted by the bacteria of the genus Brucella*.* It is a common zoonotic infection endemic to countries of the Mediterranean, Middle East, Central America, Central Asia, the Indian subcontinent, and Sub-Saharan Africa [1,2]. Transmission occurs from animals to humans by ingestion of unpasteurized dairy products, contaminated food, or exposure to tissue and fluids. Symptoms commonly present non-specifically and include fevers, chills, myalgia, nausea, vomiting, diarrhea, weight loss, and headaches. Pancytopenia represents one of the most common laboratory findings [1,2]. Brucellosis is typically not seen in developed countries, but this disease should still be considered in patients who travel to high-risk regions or suffer from atypical symptoms. We present a case of brucellosis in a patient who presented with fevers and a variety of other unusual symptoms after recently traveling to Grenada, West Indies.

## Case presentation

A 33-year-old female with no known past medical history presented to the emergency department after five days of bitemporal frontal headaches. She also reported associated photophobia, fevers, nausea, vomiting, decreased oral intake, and one day of knee pain. She denied any focal weakness, respiratory symptoms, vision change, or rash. The headaches had awoken her from sleep the night prior to admission, which prompted her visit to the emergency department. The patient had recently returned from a 10-day trip in Grenada, WI four days prior to her symptom onset. According to the patient, she was bitten by several mosquitoes while traveling in Grenada and was not exposed to any known sick contacts.

On presentation, her vital signs consisted of a temperature up to 38.1°C, blood pressure of 98/64 mmHg, heart rate of 90 beats/minute, respiratory rate of 20 breaths/minute, and oxygen saturation of 97%. Her urine pregnancy was negative and her labs were significant for white blood cell count 1.84 K/uL with bandemia of 27%, hemoglobin 11.1 g/dL, hematocrit of 34.6%, platelets 164 K/uL, and c-reactive protein (CRP) <2.9 mg/L. Basic metabolic panel was notable for sodium 134 mmol/L, potassium 3.1 mmol/L, bicarbonate 30 mmol/L, aspartate aminotransferase (AST) 31 unit/L, alanine aminotransferase (ALT) 19 unit/L, alkaline phosphatase (ALP) 55 unit/L, total bilirubin 0.5 mg/dL, and direct bilirubin 0.5mg/dL. Urinalysis and severe acute respiratory syndrome coronavirus 2 polymerase chain reaction (SARS-CoV-2 PCR) were unremarkable. CT head, chest x-ray, and EKG showed no acute findings. A lumbar puncture was performed and cerebrospinal fluid (CSF) analysis was sent (Table 1). CSF was also sent for acid-fast bacillus, bacterial blood culture with Gram stain, fungal culture, herpes simplex virus (HSV), and West Nile virus (WNV). Blood serology IgG for chikungunya, Bartonella subspecies (henselae and quintana), rickettsia IgM, IgG, and brucellosis were also sent. Syphilis screen venereal disease research laboratory (VDRL) and rapid plasma reagin (RPR) were also negative. Peripheral blood smear along with thick and thin smears was done which was normal. She was empirically started on IV fluids and oral doxycycline 100 mg twice daily while pending full infectious blood results.

**Table 1:** Cerebrospinal fluid analysis of lumbar puncture in patient case vs. various infections.

Variables	Case	Normal	Bacterial	Viral	Fungal
Opening pressure (cmH_2_O)	15	5-20	>30	Normal to mildly increased	>30
Appearance	Clear colorless	Normal	Turbid	Clear	Fibrin web
Protein (mg/dL)	23	15-40	100-100	50-100	40-300
Glucose (mg/dL)	59	50-70	<45	50-100	<45
Differential	None	None	Neutrophils	Monocytes	Monocytes
Lyme	Negative	Negative	Positive (if Lyme infection)	Negative	Negative

She continued having bitemporal headaches while admitted inpatient. However, the fevers gradually resolved and nausea slowly improved with ondansetron, and she began to tolerate food again. She had no recurrence of her previous knee pain. Her leukopenia persisted throughout her hospital stay. Serum analysis forLegionella, blood parasites, acid-fast bacilli, syphilis, fungal cultures, and blood cultures all returned negative. The patient was continued on doxycycline, and given her symptomatic improvement in her headaches and nausea, was determined stable for discharge on hospital admission day five with follow-up scheduled and send-out labs noted above still pending. Nine days post-admission, IgM anti-Brucella antibodies resulted at 3.33 (normal <0.80) with a positive IgG of 0.06 a few days later. The patient was contacted to continue the oral doxycycline 100 mg twice daily with the addition of oral rifampin 600 mg daily for a total treatment duration of six weeks. After discharge with subsequent telemedicine visits, the patient had no recurrence of symptoms. Throughout the treatment period, send-out labs were reviewed frequently and no further growth on cultures was noted. The patient completed the course of antibiotic regimen without recurrence.

## Discussion

Brucellosis is one of the most prevalent zoonotic infectious diseases in the world, affecting over 500,000 people each year globally [2]. In countries where the disease is endemic, such as the Middle East, Central and South America, Central Asia, China, Sub-Saharan Africa, and the Indian subcontinent, it is still a major cause of morbidity and mortality. Four species known to cause human brucellosis are *B. melitensis* (goats, sheep, and camels) which is most common, *B. abortus* (cows), *B. suis* (pigs), and *B. canis* (dogs). Typically, infection with* B. melitensis* and *B. suis *tends to be more virulent [1]. In endemic countries, transmission occurs from consumption of unpasteurized dairy products, contaminated meats, and exposure to tissue and fluids; however, in developed countries, infection occurs due to occupational exposure, direct skin or mucosal contact with infected livestock. While there are other Brucella species zoonosis is less likely. While incidence in the United States is only about 100-200 cases/year (which may be underreported), the number of human infections worldwide has continued to increase due to greater travel and globalization [3]. Thus, brucellosis infection must be further investigated in any patient presenting with concerning symptoms following travel to high-risk areas [1].

Even in patients without travel history, it is important to consider occupational exposures given that the primary route of bacteria transmission is by ingestion of unpasteurized dairy products. Careers such as raising livestock, butchery, farming, and veterinary medicine carry increased risk [2]. Patients presenting in spring and summer months, when brucellosis cases present most often (68%), should also be screened with increased suspicion [4]. In endemic areas, the age group between 15 years and 35 years was found to be the most frequently infected [2]. While acute symptoms of fever, arthralgia, and fatigue are among the most common in patients, headaches (the chief complaint of our patient) were seen in only 14.4% of 1098 cases studied in a retrospective evaluation by Buzgan et al. [2]. Symptoms of nausea and vomiting, such as in our patient, were observed in only 24.9% of the cases. A useful distinguishing tool in the diagnosis of brucellosis is the potential of the bacteria to manifest as febrile pancytopenia, in contrast to the classical bacteremia presentation of leukocytosis and neutrophilia with bandemia [1].

Diagnosis of brucellosis requires isolation of the bacteria such as blood cultures which may be suboptimal, bone marrow, liver tissue, lymph nodes, or other tissues to be definitive, but serologic testing is also widely used when isolation is not available [5]. The presence of IgM antibodies toBrucella, such as in our patient, indicates acute infection. However, anti-Brucella antibodies are well known to have cross-reactivity with other bacterial species such as Yersinia, *Escherichia coli *O157, *Salmonella* spp., and *Francisella tularensis* [6]. Rheumatoid factor is also a well-documented cause for false-positive IgM anti-Brucella antibodies, and so it is recommended to remove rheumatoid factor by pre-absorption beforehand [6]. When compared to IgM, Mantecón et al. found that IgG antibodies were more sensitive in the diagnosis of brucellosis, although IgG anti-Brucella antibodies have also been found to have cross-reactivity leading to false positives [7]. Although there may be false positives of IgM, clinical presentation and a proper history aid in deciding treatment. While the presence of IgM or IgG alone may provide uncertainty, the presence of both IgM and IgG tends to confirm brucellosis infection [6]. Treatment of brucellosis involves the use of a combination of doxycycline-aminoglycoside as the primary drug of choice. However, other combinations such as doxycycline-rifampin, as used in our patient, can also be considered. Treatment failure and relapse may occur in some instances with 7.8% in the doxycycline-rifampin regimen, compared to 7.4% in the doxycycline-aminoglycoside regimen. However, treatment for six to eight weeks is associated with less risk of treatment failure relapse and high therapeutic success [8-10]. In our case, we treated the patient with a combination of doxycycline-rifampin regimen for six weeks with close follow-up without recurrence of symptoms.

## Conclusions

The non-specific presentation of our patient (which included symptoms ranging from bitemporal headaches, fevers, and nausea with vomiting) demonstrates the necessity to develop a wide differential diagnosis when risk factors include travel abroad. Brucellosis, in particular, may be overlooked, given its widespread symptoms and prevalence in areas with other more common or well-known infectious diseases. For instance, our patient’s travel to Grenada where she reported mosquito bites could easily lead to greater suspicion for arbovirus illness, such as malaria, chikungunya or Zika virus. Contrastingly, patients who have eaten unpasteurized meat may not offer this information initially if they are unaware of the increased risk for brucellosis, hence history may not be supporting. In summary, while the total eradication of brucellosis in endemic areas will only be achieved by controlling its spread through animals, its identification in non-endemic countries such as the United States should be expedited by identifying those with increased risk factors, such as travel and exposures.
